# Supplementary material for: Continuous transcriptome analysis reveals novel patterns of early gene expression in Drosophila embryos
Source: Cell Genom. 2023 Feb 15;3(3):100265. doi: 10.1016/j.xgen.2023.100265 (PMC10025449; doi:10.1016/j.xgen.2023.100265)
Supplement: Document S1. Figures S1–S4, Tables S1, S2, and S4–S6, and Data S1 and S2 [file mmc1.pdf]

Cell Genomics, Volume *3*

Supplemental information

Continuous transcriptome analysis reveals novel patterns of early gene expression in *Drosophila* embryos

J. Eduardo Pérez-Mojica, Lennart Enders, Joseph Walsh, Kin H. Lau, and Adelheid Lempradl

Supplemental Information

Continuous transcriptome analysis reveals novel patterns of early gene expression.

Pérez-Mojica *et al*.

Corresponding authors: Adelheid Lempradl, Heidi.Lempradl@vai.org

This PDF file includes the following:

Figure S1..page 2
Figure S2..page 3
Figure S3..page 4
Figure S4..page 5
Data S1...page 6
Data S2…………………………………page 9
Table S1...page 13
Table S2...page 14
Table S4...page 16
Table S5...page 19
Table S6...page 20

Other supplemental material for this manuscript includes:

Table_S3.xlsx (Significantly upregulated genes during the minor Zygote Genome Activation, Related to Figure 3A. Transcripts were allocated only to the first cluster comparison to reach statistically significant differences)

Table_S7.xlsx (Normalized read counts by pseudo-time order, Related to Figure 2)

Table_S8.xlsx (Details for genes included in Table_S7, Related to Figure 2)

Table_S9.xls (Single-embryo metadata for sex-specific analysis, related to Figure 5. Single embryos were considered a replicate within a cluster (Time))

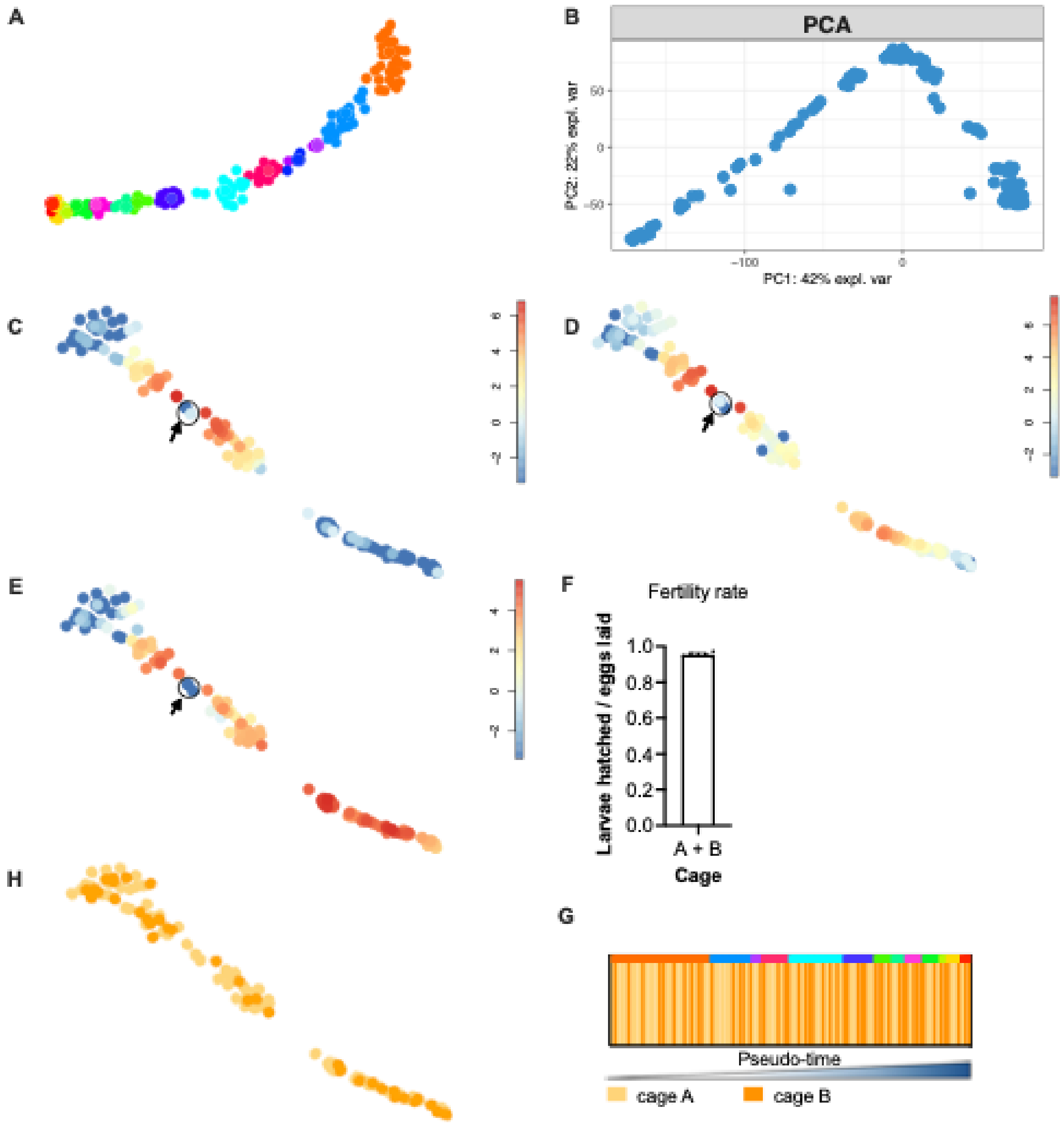

Figure S1. Alternative dimensionality reductions, identification of unfertilized eggs and analysis of batch effects in RNA-seq data, Related to Figure 1. (**A**) Fruchterman-Reingold layout or (**B**) PCA dimensionality reduction of raw RNA-seq data. (**C-E**) t-SNE maps of log2-transformed expression values with arrows and circles indicating excluded samples (n = 5) due to low levels of (**C**) *screw* (*scw*), (**D**) *scute* (*sc*) and (**E**) *escargot* (*esg*) compared to adjacent embryos on the t-SNE map and pseudo-time. (**F**) The ratio of larvae hatched from embryos taken from the two cages (A and B) used for RNA-seq on the same day of collection averaged 0.95 (n= 6, 3 replicates of ~150 embryos per cage, error bars are SD). (**H**) t-SNE map or (**G**) pseudo-time showing cage origin (A or B) of each sample.

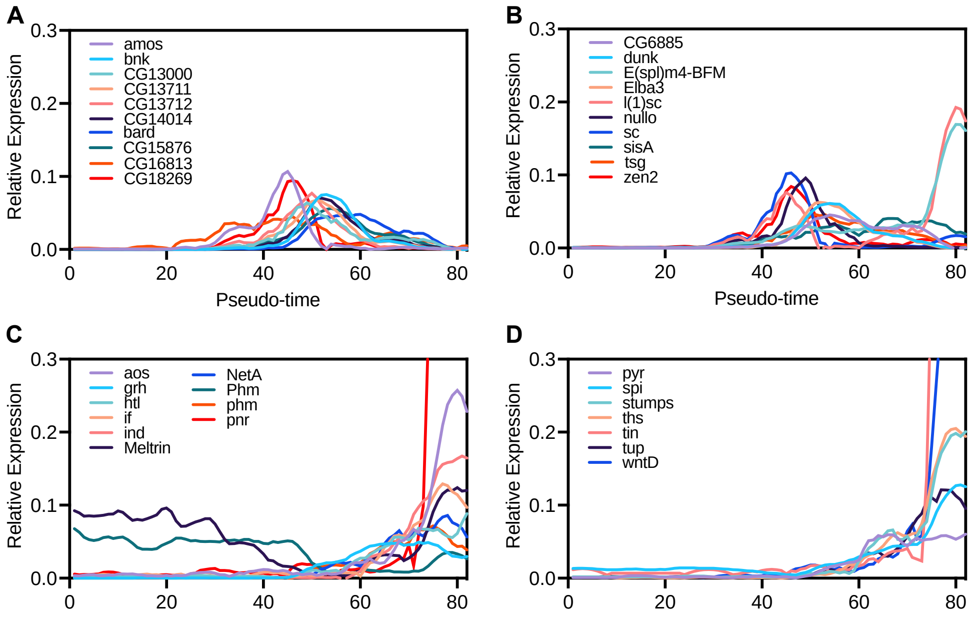

Figure S2. The continuous sequence of the ZGA, a detailed look, Related to Figure 2. Graphs show the loess-smoothed expression on reads normalized to sum 1 for (**A-B**) 20 genes reported to start transcription during NC 7-9 [S1] or (**C-D**) 17 genes reported to increase >5-fold expression from NC 14A to NC 14B [S2].

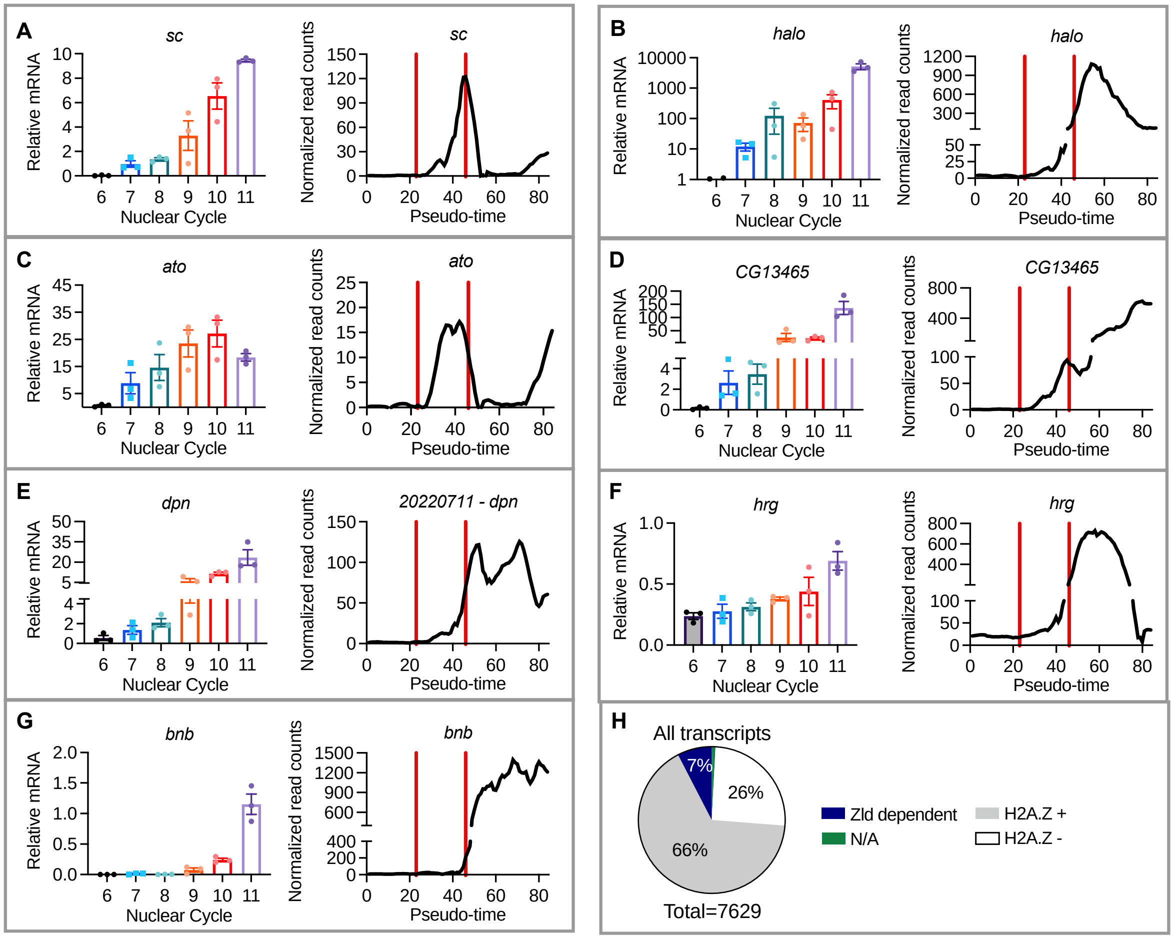

Figure S3. qPCR results for selected genes and genome distribution of Zelda or H2A.Z. (**A-G**)**, Related to Figure 3.** On the left panel, qPCR from fixed and hand-staged embryos at NC 6-11, plotted are means with SEM error bars. On the right panel, smoothed normalized reads in our pseudo-time. Normalized reads were smoothed by averaging 5 neighboring samples and a second order of the smoothing polynomial using Prism 9 version 9.4.1. Vertical red lines indicate an approximate window covering NC 6-11 in our pseudo-time. Gene symbol and gene name: *sc*, *scute*; *halo*, *halo*; *ato*, *atonal*; *CG13465*, no name; *dpn*, *deadpan*; *hrg*, *hiiragi*; *bnb*, *bangles and beads*. (**H**) Genome distribution of Zld or H2A.Z in all transcripts detected up to ~3h embryos. Zelda bounding data from Blythe and Wieschaus [S3] and H2A.Z enrichment from Ibarra-Morales *et al*. [S4]. Genes not matching between datasets are shown as N/A.

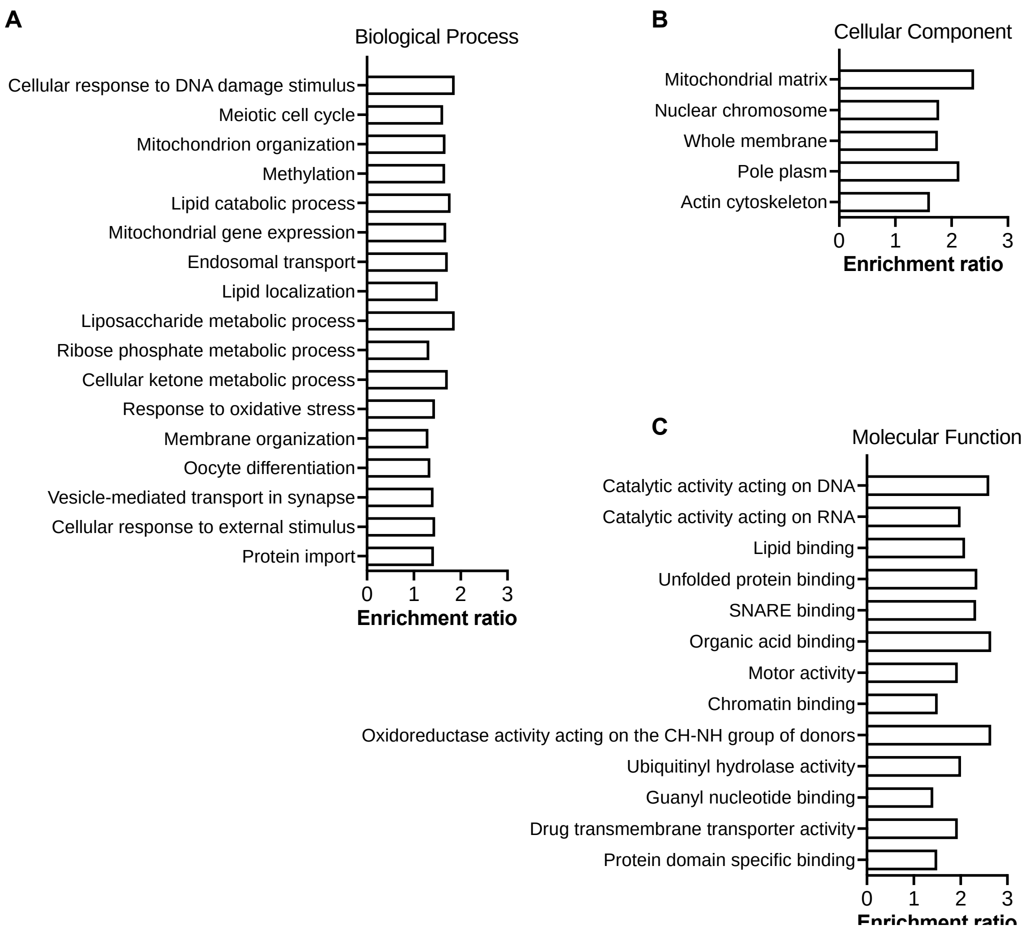

Figure S4. Pathway analysis of maternally deposited mRNAs degraded upon major ZGA. (**A-C**)**, Related to Figure 4.** ORA on all (n = 262) significantly decreased genes by comparing cluster 1 versus 5 (padj<0.01, Log2FC<-1)

Data S1

Early transcribed genes from chromosome 2

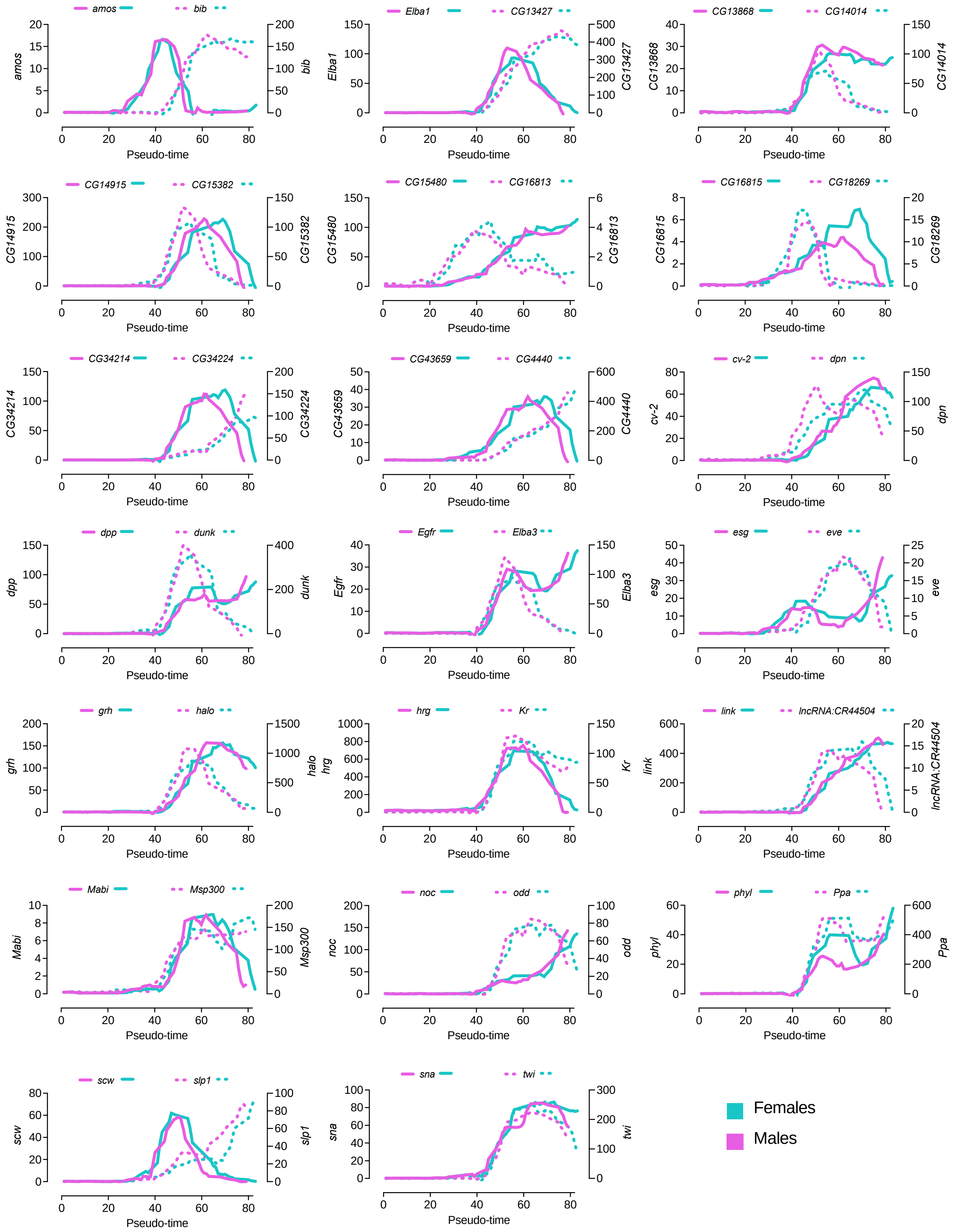

Early transcribed genes from chromosome 3

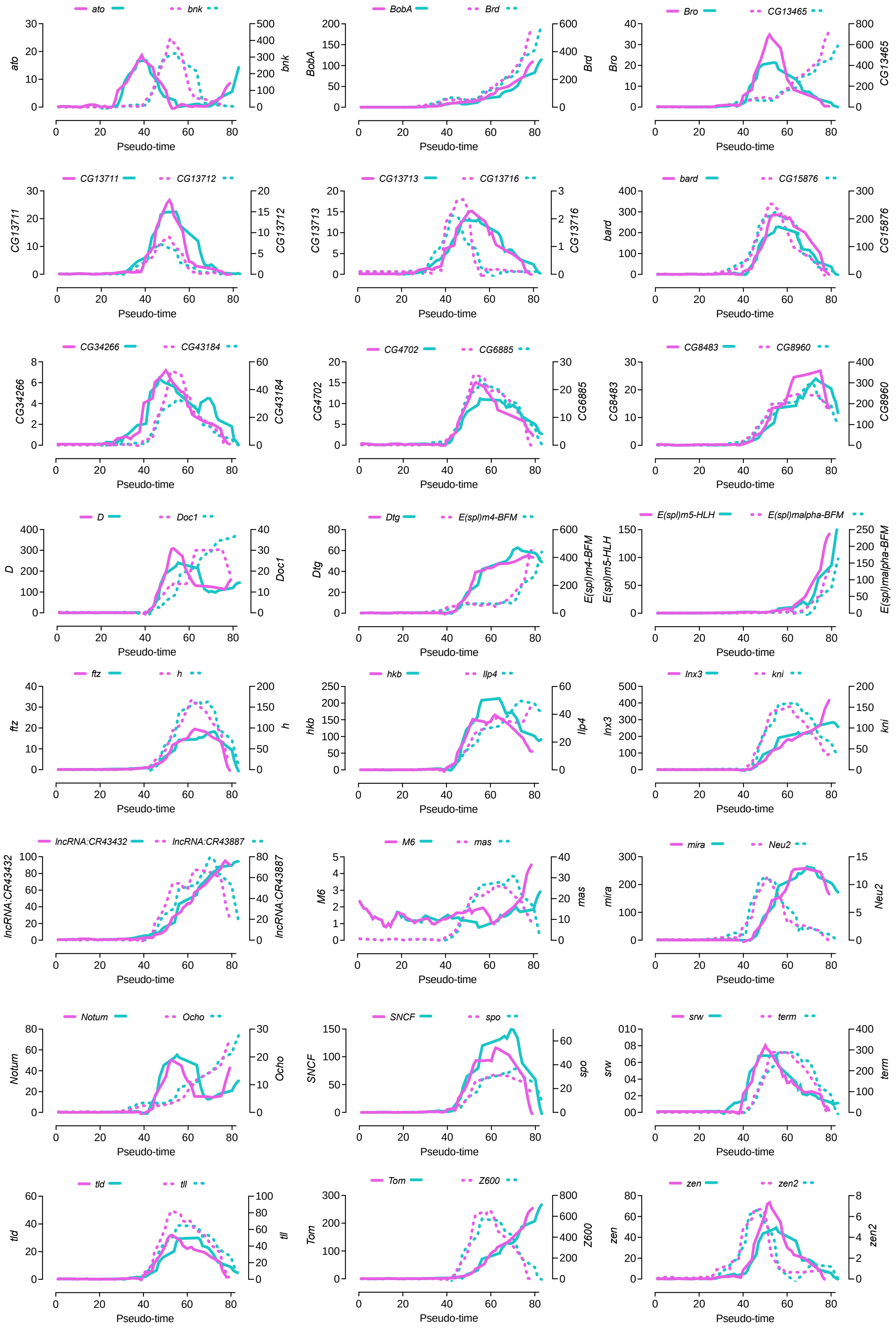

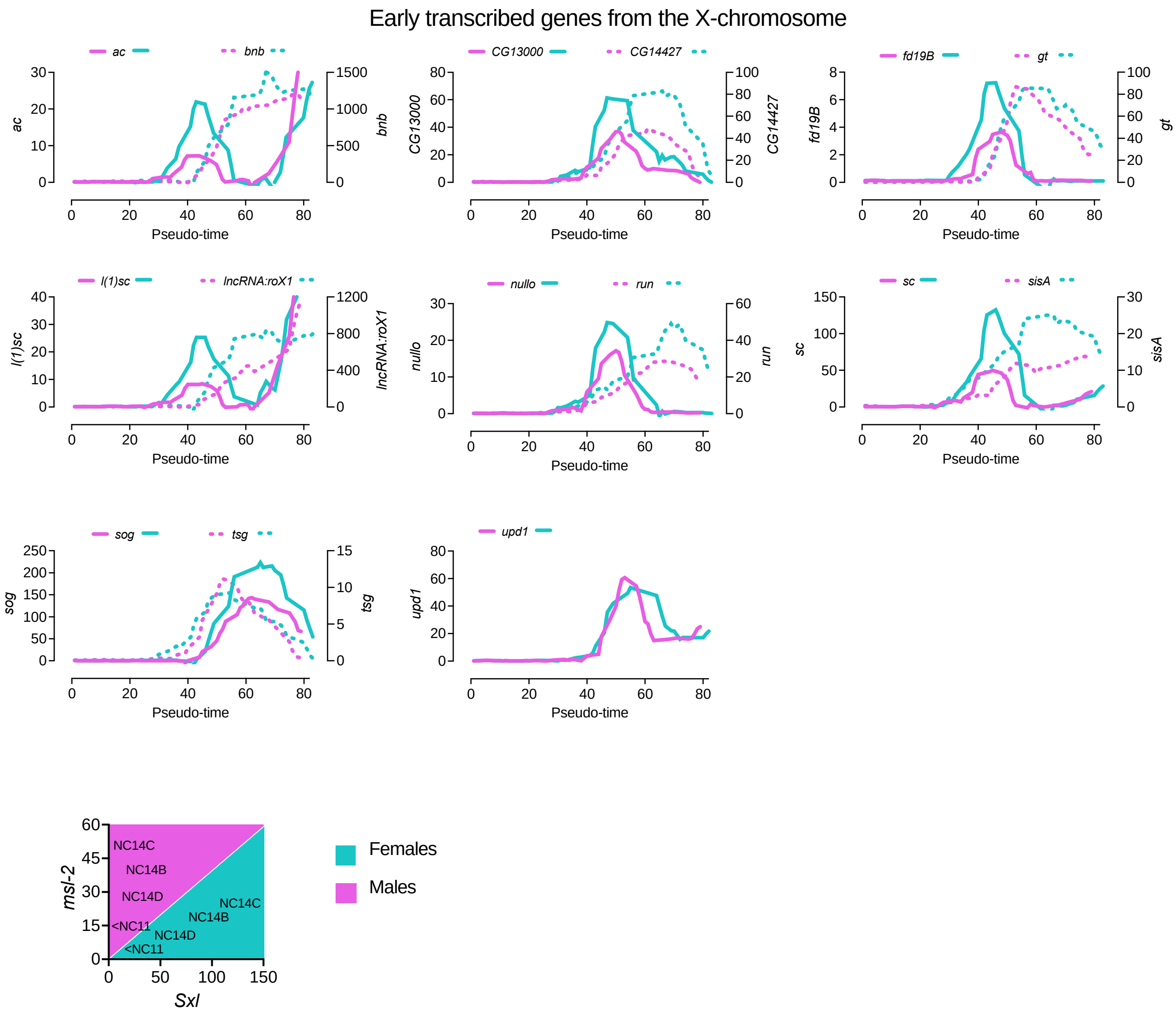

Data S1. Sex-specific transcription in early development and developed strategy to sex embryos, Related to Figure 5. (**A-C**) Smoothed normalized reads of significantly increased transcripts (padj<0.01, Log2FC>1) by comparing cluster 1 versus 2 and cluster 2 versus 3. Genes were grouped according to their genomic localization on (A) chromosome 2, (B) chromosome 3, or (C) the X-chromosome. (**D**) Cartoon showing how males and female embryos cluster by plotting *Sxl* expression on the x-axis and *msl-2* expression on the y-axis.

Data S2. Source code for this manuscript. Related to Figure 1

```
#1.- Identification of unfertilized eggs.

library(RaceID)
samples <-read.csv("01raw_reads_n192samples.txt", sep="\t", header=TRUE, row.names = 1)
sc <- SCseq(samples)
sc<-filterdata(sc, minexpr = 3, minnumber = 5, LBatch = NULL, mintotal=250000)
sc <- compdist(sc,metric="spearman", FSelect = FALSE,knn = NULL,alpha = 3)
sc <- clustexp(sc, rseed = 12345, samp = 1000 , FUNcluster = "kmedoids", verbose = F)
sc <- findoutliers(sc, probthr = 0.001, outlg = 3, outminc = 5, verbose = TRUE)

pdf(file = "01results_maps.pdf", width = 7, height = 5)
sc <- comptsne(sc,perplexity = 16, rseed = 420)
sc <- compfr(sc,knn=10)
plotmap(sc,cex=3)
plotmap(sc,cex=3,fr=TRUE)
plotlabelsmap(sc, cex = 0.2)
plotexpmap(sc, g="scw", n="scw", logsc = TRUE, cex = 3)
plotexpmap(sc, g="sc", n="sc", logsc = TRUE, cex = 3)
plotexpmap(sc, g="esg", n="esg", logsc = TRUE, cex = 3)
types <- sub("(\\_\\d+)$","", colnames(sc@ndata))
subset <- types[grep("[A]",types)]
plotsymbolsmap(sc,types,subset=subset,cex=3,leg=F,
        map=T, samples_col = rep("goldenrod1",400))
subset <- types[grep("[B]",types)]
plotsymbolsmap(sc,types,subset=subset,cex=3,leg=F,
        map=T, samples_col = rep("orange",400))
dev.off()
```

```
#2.- Comparisons with previously published data and identification of older than 3h embryos.

library(RaceID)
library(RColorBrewer)
library(FateID)
samples <-read.csv("01raw_reads_n192samples.txt", sep="\t", header=TRUE, row.names = 1)
excluded <- c("X3A.19","X3A.14","X2A.29","X2A.16","X2A.12") #identified unfertilized eggs.
samples <- samples[,!(names(samples) %in% excluded)]
sc <- SCseq(samples)
sc<-filterdata(sc, minexpr = 3, minnumber = 5, LBatch = NULL, mintotal=250000)
sc <- compdist(sc,metric="spearman", FSelect = FALSE,knn = NULL,alpha = 3)
sc <- clustexp(sc, rseed = 12345, samp = 1000 , FUNcluster = "kmedoids", verbose = F)
sc <- findoutliers(sc, probthr = 0.001, outlg = 3, outminc = 5, verbose = TRUE)

pdf(file = "02results_maps_filtered.pdf", width = 7, height = 5)
sc <- comptsne(sc,perplexity = 15, rseed = 420)
sc <- compfr(sc,knn=10)
plotmap(sc,cex=3)
plotmap(sc,cex=3,fr=TRUE)
plotlabelsmap(sc, cex = 0.2)
plotexpmap(sc, g="scw", n="scw", logsc = TRUE, cex = 3)
plotexpmap(sc, g="sc", n="sc", logsc = TRUE, cex = 3)
plotexpmap(sc, g="esg", n="esg", logsc = TRUE, cex = 3)
types <- sub("(\\_\\d+)$","", colnames(sc@ndata))
subset <- types[grep("[A]",types)]
plotsymbolsmap(sc,types,subset=subset,cex=3,leg=F,
        map=T, samples_col = rep("goldenrod1",400))
subset <- types[grep("[B]",types)]
plotsymbolsmap(sc,types,subset=subset,cex=3,leg=F,
        map=T, samples_col = rep("orange",400))
dev.off()

clusters <- sc@cpart
write.csv(clusters, file = "02results_clusters_filtered.csv")

pdf(file = "02results_lineage_analysis_filtered.pdf", width = 7, height = 5)
ltr <- Ltree(sc)
ltr <- compentropy(ltr)
ltr <- projcells(ltr,cthr=1,nmode=T,knn=3)
```

```
ltr <- projback(ltr,pdishuf = 100, fast=FALSE, rseed=17000)
ltr <- lineagegraph(ltr)
ltr <- comppvalue(ltr,pthr=0.05, sensitive = T)
x <- compscore(ltr)
plotspantree(ltr,cex = 3,projections = T)
dev.off()

n <- cellsfromtree(ltr,c(2,8,9,5,7,13,3,6,1,4,12,10,11)) #select pseudo-temporal order vector from StemID.
x <- getfdata(ltr@sc)
fs  <- filterset(x,n=n$f, minexpr = 0, minnumber = 0) #additional filtering and subsetting of gene expression
y    <- ltr@sc@cpart[n$f]
length(y)
fcol <- ltr@sc@fcol
write.table(y,"02results_pseudotime_names_filtered.csv",col.names=TRUE,sep=",",quote=FALSE)

pdf(file="02results_Kwasnieski_and_Sandler_filtered.pdf",width = 7, height = 5)
COL<-brewer.pal(n=12,name="Set3")
plotexpmap(sc, logsc = TRUE, cex = 3,
           g=c("E(spl)m4-BFM","Elba3","l(1)sc","bnk","amos","dunk","nullo","sisA","sc",
               "tsg","zen2","CG13000","CG13711","CG13712","CG14014","bard","CG15876",
               "CG18269","CG6885","CG16813"),
           n="List from Kwasnieski et al., 2019 (NC7-9)")
plotexpression(fs, y, n$f, col = fcol, alpha=.2, types=NULL, ylab = "Normalized Read Counts",
               g=c("E(spl)m4-BFM","Elba3","l(1)sc","bnk","amos","dunk","nullo","sisA","sc",
                   "tsg","zen2","CG13000","CG13711","CG13712","CG14014","bard","CG15876",
                   "CG18269","CG6885","CG16813"),
               cluster=FALSE, logsc = F,name = "List from Kwasnieski et al., 2019 (NC7-9)")
plotexpressionProfile(fs, y, n$f, col = COL, alpha=.2, lwd=5, ylim = c(0,0.1), ylab = "Normalized Expression",
                      g=c("E(spl)m4-BFM","Elba3","l(1)sc","bnk","amos","dunk","nullo","sisA","sc",
                          "tsg","zen2","CG13000","CG13711","CG13712","CG14014","bard","CG15876",
                          "CG18269","CG6885","CG16813"),
                      cluster=FALSE, name="List from Kwasnieski et al., 2019 (NC7-9)")
plotexpmap(sc, logsc = TRUE, cex = 3,
           g=c("stumps", "ush", "Meltrin", "if", "ind", "aos", "ths",
               "pnr", "spi", "pyr", "grh", "phm", "wntD", "tin",
               "htl", "tup", "NetA"),
           n="List from Sandler and Stathopoulos, 2016 - (14A to 14B)")
plotexpression(fs, y, n$f, col = fcol, alpha=.2, types=NULL, ylab = "Normalized Read Counts",
               g=c("stumps", "ush", "Meltrin", "if", "ind", "aos", "ths",
                   "pnr", "spi", "pyr", "grh", "phm", "wntD", "tin",
                   "htl", "tup", "NetA"),
               cluster=FALSE, logsc = F,name ="List from Sandler and Stathopoulos, 2016 - (14A to 14B)")
plotexpressionProfile(fs, y, n$f, col = COL, alpha=.2, lwd=5, ylim = c(0,0.07), ylab = "Normalized Expression",
                      g=c("stumps", "ush", "Meltrin", "if", "ind", "aos", "ths",
                          "pnr", "spi", "pyr", "grh", "phm", "wntD", "tin",
                          "htl", "tup", "NetA"),
                      cluster=FALSE, name="List from Sandler and Stathopoulos, 2016 - (14A to 14B)")
dev.off()
```

#3.- Generation of pseudo-time using only 3h embryos and differential expression analysis.

```
library(RaceID)
library(RColorBrewer)
library(FateID)
samples <-read.csv("01raw_reads_n192samples.txt", sep="\t", header=TRUE, row.names = 1)
excluded <- c("X3A.19","X3A.14","X2A.29","X2A.16","X2A.12", #identified unfertilized eggs.
          "X1A.04","X1B.22","X3B.05","X3B.22","X3A.27", #cluster 3 (older than 3h)
          "X1B.17","X3A.21","X1A.28","X1B.28","X3B.28", #cluster 6 (older than 3h)
          "X1A.01", "X1B.24","X1B.29", "X1B.31","X3B.25","X3B.27", #cluster 1 (older than 3h)
          "X1A.17","X1A.18","X1B.23","X2B.07","X3A.08", #cluster 4 (older than 3h)
          "X2B.03","X3B.23", #cluster 12 (older than 3h)
          "X2A.22","X3A.24","X1A.14","X2A.10","X2B.30", #cluster 10 (older than 3h)
          "X2B.02", "X2B.25") #cluster 11 (older than 3h)
samples <- samples[,!(names(samples) %in% excluded)]
sc <- SCseq(samples)
sc<-filterdata(sc, minexpr = 3, minnumber = 5, LBatch = NULL, mintotal=250000)
sc <- compdist(sc,metric="spearman", FSelect = FALSE,knn = NULL,alpha = 3)
sc <- clustexp(sc, rseed = 12345, samp = 1000 , FUNcluster = "kmedoids", verbose = F)
```

```
sc <- findoutliers(sc, probthr = 0.001, outlg = 3, outminc = 5, verbose = TRUE)

pdf(file = "03results_maps_filtered_3h.pdf", width = 7, height = 5)
sc <- comptsne(sc,perplexity = 11.5, rseed = 1234) #13.659 #10.5 #11.45 #11.2
sc <- compfr(sc,knn=10)
plotmap(sc,cex=3)
plotmap(sc,cex=3,fr=TRUE)
plotlabelsmap(sc, cex = 0.2)
plotexpmap(sc, g="scw", n="scw", logsc = TRUE, cex = 3)
plotexpmap(sc, g="sc", n="sc", logsc = TRUE, cex = 3)
plotexpmap(sc, g="esg", n="esg", logsc = TRUE, cex = 3)
types <- sub("(\\_\\d+)$","", colnames(sc@ndata))
subset <- types[grep("[A]",types)]
plotsymbolsmap(sc,types,subset=subset,cex=3,leg=F, map=T, samples_col = rep("goldenrod1",400))
subset <- types[grep("[B]",types)]
plotsymbolsmap(sc,types,subset=subset,cex=3,leg=F, map=T, samples_col = rep("orange",400))
dev.off()

pdf(file = "03results_stemID_cell_projection_filtered_3h.pdf", width = 7, height = 5)
ltr <- Ltree(sc) #construct object used for StemID analysis
ltr <- compentropy(ltr)  #calculation of the transcriptome entropy of each cell
ltr <- projcells(ltr,cthr=1,nmode=T,knn=3)  #cell projection calculation,cthr=1,nmode=TRUE,knn=3
ltr<-projback(ltr,pdishuf = 100, fast=FALSE, rseed=17000)
ltr <- lineagegraph(ltr) #lineage tree is inferred
ltr <- comppvalue(ltr,pthr=0.05, sensitive = T)  #p-values for the links calculation
x <- compscore(ltr)
plotspantree(ltr,cex = 3,projections = T)
dev.off()

n <- cellsfromtree(ltr,c(1,5,4,6,2,9,3,7)) #select pseudo-temporal order vector from StemID.
x <- getfdata(ltr@sc)
fs  <- filterset(x,n=n$f, minexpr = 0, minnumber = 0) #additional filtering and subsetting of gene expression
y    <- ltr@sc@cpart[n$f]
length(y)
fcol <- ltr@sc@fcol
write.table(y,"03results_pseudotime_order_filtered_3h.csv",col.names=TRUE,sep=",",quote=FALSE)
yy <- as.data.frame(y) #y is an integer and I don't know how to get rownames from it so I convert it into a data frame
list_pseudotime <- row.names(yy)
norm_counts <- as.matrix(getfdata(sc))
norm_counts <- norm_counts[, list_pseudotime] #to get counts on pseudotemporal order
write.csv(norm_counts, file = "03results_fateid_counts_ps_norm_filtered_3h.csv")

A <- names(sc@cpart)[sc@cpart %in% c(1)]
B <- names(sc@cpart)[sc@cpart %in% c(5)]
x <- diffexpnb(sc@expdata,n=c(A,B),DESeq = TRUE, A=A, B=B , method = "per-condition")
write.table(x$res,"03diffexpnb_1_cl1and5_filtered_3h.xls",col.names=TRUE,sep="\t",quote=FALSE)
A <- names(sc@cpart)[sc@cpart %in% c(5)]
B <- names(sc@cpart)[sc@cpart %in% c(4)]
x <- diffexpnb(sc@expdata,n=c(A,B),DESeq = TRUE, A=A, B=B , method = "per-condition")
write.table(x$res,"03diffexpnb_2_cl5and4_filtered_3h.xls",col.names=TRUE,sep="\t",quote=FALSE)
A <- names(sc@cpart)[sc@cpart %in% c(4)]
B <- names(sc@cpart)[sc@cpart %in% c(6)]
x <- diffexpnb(sc@expdata,n=c(A,B),DESeq = TRUE, A=A, B=B , method = "per-condition")
write.table(x$res,"03diffexpnb_3_cl4and6_filtered_3h.xls",col.names=TRUE,sep="\t",quote=FALSE)
A <- names(sc@cpart)[sc@cpart %in% c(6)]
B <- names(sc@cpart)[sc@cpart %in% c(2)]
x <- diffexpnb(sc@expdata,n=c(A,B),DESeq = TRUE, A=A, B=B , method = "per-condition")
write.table(x$res,"03diffexpnb_4_cl6and2_filtered_3h.xls",col.names=TRUE,sep="\t",quote=FALSE)
A <- names(sc@cpart)[sc@cpart %in% c(1)]
B <- names(sc@cpart)[sc@cpart %in% c(2)]
x <- diffexpnb(sc@expdata,n=c(A,B),DESeq = TRUE, A=A, B=B , method = "per-condition")
write.table(x$res,"03diffexpnb_5_cl1and2_filtered_3h.xls",col.names=TRUE,sep="\t",quote=FALSE)
```

#4.- Differential expression analysis between males and females in 3h embryos. Input data can be found in Supplemental_Table_S6.xlsx

```
library(splineTimeR)
library(Biobase)

read.counts <-read.csv("normalized_reads.csv", sep=",", header=TRUE, row.names = 1)
psinfo_table <- read.csv("metadata.csv", sep=",", header=TRUE)
psnames <- psinfo_table[,1]
pssamples <- read.counts[,psnames]
row.names(psinfo_table) <- colnames(pssamples)
psinfo_table$SampleName <- factor(psinfo_table$SampleName)
psinfo_table$Treatment <- factor(psinfo_table$Treatment)
psinfo_table$Replicate <- factor(psinfo_table$Replicate)
all(rownames(psinfo_table) %in% colnames(pssamples))
all(rownames(psinfo_table) == colnames(pssamples))

phenoData <- new("AnnotatedDataFrame", data=psinfo_table)
minimalSet <- ExpressionSet(assayData=as.matrix(pssamples), phenoData = phenoData)
diffExprs <- splineDiffExprs(eSetObject = minimalSet, df = 7,
                cutoff.adj.pVal = 0.01, reference = "MALE",
                intercept = TRUE)
write.csv(diffExprs, file = "04results_7df_q0.01.csv")
```

Table S1. Previously reported genes during the minor and major ZGA, Related to Figure 2. Genes transcribed during the minor ZGA were taken from Kwasnieski et al. [S5]. Genes transcribed during the major ZGA were taken from Sandler and Stathopoulos [S2].

Minor ZGA (NC 7-9)		Major ZGA (NC 14A-14B)	
FlybaseID	Gene symbol	FlybaseID	Gene symbol
FBgn0002629	E(spl)m4-BFM	FBgn0020299	stumps
FBgn0031621	Elba3	FBgn0003963	ush
FBgn0002561	l(1)sc	FBgn0265140	Meltrin
FBgn0004389	bnk	FBgn0001250	if
FBgn0003270	amos	FBgn0025776	ind
FBgn0083973	dunk	FBgn0004569	aos
FBgn0004143	nullo	FBgn0033652	ths
FBgn0003411	sisA	FBgn0003117	pnr
FBgn0004170	sc	FBgn0005672	spi
FBgn0003865	tsg	FBgn0033649	pyr
FBgn0004054	zen2	FBgn0259211	grh
FBgn0030807	CG13000	FBgn0004959	phm
FBgn0035572	CG13711	FBgn0038134	wntD
FBgn0035570	CG13712	FBgn0004110	tin
FBgn0031718	CG14014	FBgn0010389	htl
FBgn0038566	bard	FBgn0003896	tup
FBgn0035569	CG15876	FBgn0015773	NetA
FBgn0031719	CG18269	N/A	N/A
FBgn0036810	CG6885	N/A	N/A
FBgn0032490	CG16813	N/A	N/A

Table S2. Pseudo-time order, cluster number, sample ID and sex for each embryo, Related to Figure 5

Pseudo-time order	Cluster	Sample ID	Sex	Pseudo-time order	Cluster	Sample ID	Sex
1	1	X1A.22	NO DNA	43	3	X2A.04	FEMALE
2	1	X1B.04	NO DNA	44	3	X2A.32	FEMALE
3	1	X2A.07	NO DNA	45	3	X2A.30	MALE
4	1	X1B.20	NO DNA	46	3	X2A.15	MALE
5	1	X1A.19	NO DNA	47	3	X2B.20	FEMALE
6	1	X1A.02	NO DNA	48	4	X2A.21	FEMALE
7	1	X1A.20	NO DNA	49	4	X2A.18	MALE
8	1	X1A.25	NO DNA	50	4	X2B.24	FEMALE
9	1	X1A.11	NO DNA	51	5	X3B.04	MALE
10	1	X1B.03	NO DNA	52	5	X3A.03	MALE
11	1	X1B.13	NO DNA	53	5	X3A.11	MALE
12	1	X1A.12	NO DNA	54	5	X3B.03	MALE
13	1	X1B.15	NO DNA	55	5	X3B.15	FEMALE
14	1	X1A.05	NO DNA	56	5	X3A.28	FEMALE
15	1	X1A.13	NO DNA	57	5	X3A.25	FEMALE
16	1	X2A.06	NO DNA	58	5	X1B.05	MALE
17	1	X1A.07	NO DNA	59	5	X3B.01	MALE
18	1	X1A.15	NO DNA	60	6	X3B.02	MALE
19	1	X1B.09	NO DNA	61	6	X3B.19	MALE
20	1	X1A.32	NO DNA	62	6	X3A.31	MALE
21	1	X2B.04	NO DNA	63	6	X3A.09	MALE
22	1	X1A.09	NO DNA	64	6	X3B.06	MALE
23	1	X1B.27	FEMALE	65	7	X3B.20	FEMALE
24	1	X1A.24	MALE	66	6	X3A.23	FEMALE
25	1	X2A.20	MALE	67	6	X2B.09	FEMALE
26	1	X2A.24	MALE	68	7	X3B.30	FEMALE

27	1	X1A.27	MALE	69	7	X2B.19	MALE
28	1	X1B.11	NO DNA	70	7	X2A.25	FEMALE
29	1	X2B.06	FEMALE	71	7	X2A.08	FEMALE
30	2	X2A.17	FEMALE	72	7	X2A.03	MALE
31	2	X2B.26	FEMALE	73	7	X3A.30	FEMALE
32	2	X2A.27	MALE	74	7	X3B.31	FEMALE
33	2	X2A.23	FEMALE	75	7	X2A.19	FEMALE
34	2	X2A.26	MALE	76	7	X1B.10	MALE
35	2	X2A.11	MALE	77	8	X3B.11	MALE
36	2	X2A.28	FEMALE	78	8	X3A.12	MALE
37	2	X2B.28	FEMALE	79	8	X1A.31	MALE
38	2	X2B.12	MALE	80	8	X3B.29	MALE
39	2	X2B.18	MALE	81	8	X3B.26	FEMALE
40	2	X2B.21	MALE	82	8	X3A.07	FEMALE
41	3	X2B.15	FEMALE	82	8	X1B.12	FEMALE
42	1	X1A.22	NO DNA	84	8	X2B.22	FEMALE

Table S4. Transcripts with significantly different expression in male versus female embryos during the first 3h of development, Related to Figure 5.

FlybaseID	Gene symbol	Chr	FlybaseID	Gene symbol	Chr
FBgn0264442	ab	2L	FBgn0030289	GCS1	X
FBgn0000117	arm	X	FBgn0020300	geko	3L
FBgn0000137	ase	X	FBgn0027287	Gmap	X
FBgn0002069	AspRS	2R	FBgn0037376	Hat1	3R
FBgn0030960	Atg101	X	FBgn0040318	HGTX	3L
FBgn0004862	bap	3R	FBgn0003997	hid	3L
FBgn0288686	betaTub60D	2R	FBgn0004828	His3.3B	X
FBgn0050362	boly	2R	FBgn0027596	Kank	2R
FBgn0000229	bsk	2L	FBgn0261955	kdn	X
FBgn0029957	CG12155	X	FBgn0013469	klu	3L
FBgn0030420	pira	X	FBgn0267348	LanB2	3L
FBgn0029771	CG12730	X	FBgn0262109	lncRNA:CR42862	3L
FBgn0030868	CG12986	X	FBgn0267665	lncRNA:CR46003	3L
FBgn0030807	CG13000	X	FBgn0261260	mgl	X
FBgn0035186	CG13912	3L	FBgn0036486	Msh6	3L
FBgn0029931	CG14427	X	FBgn0005616	msl-2	2L
FBgn0031632	CG15628	2L	FBgn0030766	mthl1	X
FBgn0029990	CG2233	X	FBgn0002873	mud	X
FBgn0023526	CG2865	X	FBgn0016684	NaPi-T	2R
FBgn0023529	Grp170	X	FBgn0030605	ND-B18	X
FBgn0028491	CG2930	X	FBgn0030500	Ndc80	X
FBgn0040373	CG3038	X	FBgn0260011	NimC4	2L
FBgn0053494	CG33494	3R	FBgn0015777	nrv2	2L
FBgn0085322	CG34293	3R	FBgn0039678	Obp99a	3R
FBgn0085430	Dora	X	FBgn0015524	otp	2R

FBgn0034958	CG3907	2R	FBgn0004394	pdm2	2L
FBgn0037801	CG3999	3R	FBgn0027621	Pfrx	X
FBgn0025627	CG4194	X	FBgn0013725	phyl	2R
FBgn0259244	CG42342	3R	FBgn0025739	pon	X
FBgn0263776	CG43693	3L	FBgn0283741	prage	X
FBgn0264711	CG43980	3L	FBgn0267507	pre-rRNA:CR45847	rDNA
FBgn0030796	CG4829	X	FBgn0083940	RhoU	X
FBgn0031913	CG5958	2L	FBgn0005631	robo1	2R
FBgn0038407	CG6126	3R	FBgn0003300	run	X
FBgn0030990	CG7556	X	FBgn0003302	rux	X
FBgn0031001	CG7884	X	FBgn0004170	sc	X
FBgn0030863	CG8188	X	FBgn0025682	scf	3L
FBgn0030854	CG8289	X	FBgn0004880	scrt	3L
FBgn0034479	CG8654	2R	FBgn0003345	sd	X
FBgn0036381	CG8745	3L	FBgn0261873	sdt	X
FBgn0030514	Mrgn1	X	FBgn0003411	sisA	X
FBgn0029504	CHES-1-like	X	FBgn0026179	siz	3L
FBgn0263257	Cngl	X	FBgn0039873	Smvt	3R
FBgn0030028	Corp	X	FBgn0003463	sog	X
FBgn0025641	DAAM	X	FBgn0036411	Sox21a	3L
FBgn0020493	Dad	3R	FBgn0020378	Sp1	X
FBgn0263930	dally	3L	FBgn0260440	spdo	3R
FBgn0260635	Diap1	3L	FBgn0037684	Srr	3R
FBgn0020307	dve	2R	FBgn0003459	stwl	3L
FBgn0260400	elav	X	FBgn0263755	Su(var)3-9	3R
FBgn0013953	Esp	3R	FBgn0040271	Sulf1	3R
FBgn0038665	euc	3R	FBgn0264270	Sxl	X
FBgn0000320	eya	2L	FBgn0017482	T3dh	2R
FBgn0000635	Fas2	X	FBgn0267001	Ten-a	X

FBgn0004898	fd96Cb	3R	FBgn0019650	toy	4
FBgn0030092	fh	X	FBgn0046687	Tre1	X
FBgn0000658	fj	2R	FBgn0035521	VhaM9.7-a	3L
FBgn0037724	Fst	3R	FBgn0086680	vvl	3L
FBgn0016797	fz2	3L	FBgn0010453	Wnt4	2L
FBgn0038391	GATAe	3R	FBgn0001983	wor	2L

Chr, Chromosome

Table S5. Summary stats of RNA-seq data. Related to Figure 1

Parameter	Library 1	Library 2
Number of Reads	623742873	651920184
Reads With Valid Barcodes	0.966314	0.965711
Sequencing Saturation	0.894705	0.884992
Q30 Bases in CB+UMI	0.96923	0.969831
Q30 Bases in RNA read	0.928618	0.93717
Reads Mapped to Genome: Unique+Multiple	0.940993	0.953845
Reads Mapped to Genome: Unique	0.91538	0.927777
Reads Mapped to Transcriptome: Unique+Multipe Genes	0.819441	0.841894
Reads Mapped to Transcriptome: Unique Genes	0.799854	0.819808
Estimated Number of Cells	68	64
Reads in Cells Mapped to Unique Genes	495767614	530205406
Fraction of Reads in Cells	0.993714	0.992059
Mean Reads per Cell	7290700	8284459
Median Reads per Cell	6484011	8574043
UMIs in Cells	51419909	60143358
Mean UMI per Cell	756175	939739
Median UMI per Cell	684763	937929
Mean Genes per Cell	7473	7487
Median Genes per Cell	7279	7285
Total Genes Detected	12842	12866

Each library included 96 barcoded single embryos.

Table S6. Primer sequences used in qPCR experiments and amplicon size in base pairs. Related to Figure S3

FlybaseID or Chr location	Gene symbol	Forward sequence (Fwd | 5'-3')<br>Reverse sequence (Rev | 5'-3')	Amplicon size (bp)
FBgn0040809	CG13465	Fwd | GCAGATCATCAGTAACCAACCTG<br>Rev | GAAACAAGAGCGGTTTCGGCA	53
FBgn0010433	ato	Fwd | AAACGATTCCAGCCTCAGCA<br>Rev | TCATCGAACAAGGCGGAGTT	175
FBgn0010109	dpn	Fwd | ACGACGATTTCGACTGCTCC<br>Rev | CCATAATCGGTTTGTTGGTCTTTCT	117
FBgn0001174	halo	Fwd | GCACTGCACTCTGGACACTC<br>Rev | TGGAAAAAGTAGCTCGGCCA	104
FBgn0001090	bnb	Fwd | GTGCGTTGTGTGTTTTGTTGC<br>Rev | GCAGTTGGCACTTCATTCTTTTG	179
FBgn0015949	hrg	Fwd | CGGCACCGACAATTTCTTTGTA<br>Rev | AGGCATCAATGCCGAGGAAA	101
FBgn0004170	sc	Fwd | CCAACGACCATCAATTCGGC<br>Rev | CGAGGAACCAGGCGATAGAG	195
FBgn0036058	CG6707	Fwd | CAGAGTACCAACACCGGAGG<br>Rev | GAGCGTTCCGAATGGGAGTA	136
FBgn0023517	Pgam5	Fwd | GGCATCAAGTGGGACAAGGT<br>Rev | CGTCGCGAAGGAAAGATGC	190
ChrX:4016153..4016261	N/A	Fwd | TTGGGCTGCTTCAGGTTTGA<br>Rev | GAAGAGACACGCCAAGGCTA	109
ChrY:200524..200676	N/A	Fwd | TCATAAGGAGTGAAGCGGTCC<br>Rev | AATTGTGTGCATCGGTGGGTC	153

All primers were designed and optimized in the lab. bp, base pairs; Chr, chromosome; N/A, not applicable

REFERENCES

[S1] Lott, S.E., Villalta, J.E., Schroth, G.P., Luo, S., Tonkin, L.A., and Eisen, M.B. (2011). Noncanonical Compensation of Zygotic X Transcription in Early Drosophila melanogaster Development Revealed through Single-Embryo RNA-Seq. PLoS Biology *9*, e1000590. 10.1371/journal.pbio.1000590.

[S2] Sandler, J.E., and Stathopoulos, A. (2016). Quantitative Single-Embryo Profile of Drosophila Genome Activation and the Dorsal-Ventral Patterning Network. Genetics *202*, 1575–1584.

[S3] Blythe, S.A., and Wieschaus, E.F. (2015). Zygotic genome activation triggers the DNA replication checkpoint at the midblastula transition. Cell *160*, 1169–1181.

[S4] Ibarra-Morales, D., Rauer, M., Quarato, P., Rabbani, L., Zenk, F., Schulte-Sasse, M., Cardamone, F., Gomez-Auli, A., Cecere, G., and Iovino, N. (2021). Histone variant H2A.Z regulates zygotic genome activation. Nat. Commun. *12*, 7002.

[S5] Kwasnieski, J.C., Orr-Weaver, T.L., and Bartel, D.P. (2019). Early genome activation in Drosophila is extensive with an initial tendency for aborted transcripts and retained introns. Genome Res. *29*, 1188–1197.
